# Optimizing fault tolerance of RAM cell through MUX based modeling and design using symmetries of QCA cells

**DOI:** 10.1038/s41598-024-59185-2

**Published:** 2024-04-13

**Authors:** Syed Farah Naz, Suhaib Ahmed, Shafqat Nabi Mughal, Mohammed Asger, Jadav Chandra Das, Saurav Mallik, Mohd Asif Shah

**Affiliations:** 1 Department of Electrical Engineering, Indian Institute of Technology, Jammu, India; 2 Department of Electronics and Communication Engineering, Model Institute of Engineering and Technology, Jammu, J&K India; 3 Department of Electrical Engineering, BGSB University, Rajouri, India; 4 School of Material Sciences and Nanotechnology, BGSB University, Rajouri, India; 5 Department of Information Technology, Maulana Abul Kalam Azad University of Technology, Haringhata, West Bengal, Nodia India; 6 Department of Environmental Health, Harvard T H Chan School of Public Health, Boston, MA 02115 USA; 7 Department of Economics, Kebri Dehar University, 250 Kebri Dehar, Somali Ethiopia; 8 Division of Research and Development, Lovely Professional University, Phagwara, Punjab 144001 India; 9 Centre for Research Impact and Outcome, Chitkara University, Rajpura, Punjab 140401 India

**Keywords:** Random Access Memory, Quantum dot Cellular Automata, Quantum Cells, Fault Tolerant Design, Nanoelectronics, Multiplexer, Engineering, Nanoscience and technology, Physics

## Abstract

Extensive research is now being conducted on the design and construction of logic circuits utilizing quantum-dot cellular automata (QCA) technology. This area of study is of great interest due to the inherent advantages it offers, such as its compact size, high speed, low power dissipation, and enhanced switching frequency in the nanoscale domain. This work presents a design of a highly efficient RAM cell in QCA, utilizing a combination of a 3-input and 5-input Majority Voter (MV) gate, together with a 2 × 1 Multiplexer (MUX). The proposed design is also investigated for various faults such as single cell deletion, single cell addition and single cell displacement or misalignment defects. The circuit under consideration has a high degree of fault tolerance. The functionality of the suggested design is showcased and verified through the utilization of the QCADesigner tool. Based on the observed performance correlation, it is evident that the proposed design demonstrates effectiveness in terms of cell count, area, and latency. Furthermore, it achieves a notable improvement of up to 76.72% compared to the present configuration in terms of quantum cost. The analysis of energy dissipation, conducted using the QCAPro tool, is also shown for various scenarios. It is seen that this design exhibits the lowest energy dispersion, hence enabling the development of ultra-low power designs for diverse microprocessors and microcontrollers.

## Introduction

The attempts to develop smaller and more energy-efficient devices using Complementary Metal Oxide Semiconductors (CMOS) have exposed critical constraints of the CMOS technology: short channel effects and significant leakage capacity. Many quantum mechanical effects appear in CMOS technology that can’t be obviated. Alternatives to CMOS as presented are Carbon Nanotube Field Effect Transistors^1–3^, Nano-wire based Transistor^4,5^ and Quantum-dot Cellular Automata (QCA)^6–8^. QCA is highlighted over others because of its speedy operation, speed and low-power dissipation. The quantum behavior of the electrons in quantum dots^9–11^ is utilized by QCA, a computing paradigm based on nanotechnology, to carry out computations. Symmetry is essential to QCA because it facilitates the design and comprehension of QCA circuits, reduces mistakes, and increases information processing effectiveness. The following are some fundamental ideas and uses of symmetry in quantum dot cellular automata:

i. *Circuit design:* Designing effective and dependable QCA circuits can be made easier by comprehending the symmetries of QCA arrays. The design of functional units and logic gates can be guided by symmetry considerations, which can also assist in spotting regular patterns in the arrangement of quantum dots.
ii. *Error reduction:* In QCA devices, symmetry can be used to reduce errors. It is feasible to inhibit some error channels by constructing systems with particular symmetries, improving the overall reliability of the QCA computation.
iii. *State preparation and initialization:* To design QCA systems that are more stable and simpler to initialize into desirable states, symmetry can be used. Robustness against disturbances during the startup procedure may result from specific symmetries.
iv. *Signal propagation:* Signal propagation in QCA arrays is governed by symmetry considerations. Designing effective channels for information transfer and signal processing can be aided by understanding the symmetries of the system.

Hence, by utilizing symmetries, we can create more reliable circuits, reduce errors, manage signal propagation, and investigate special features for quantum information processing and computation.

QCA technology has been used to design different logics like adders^12–15^, switching networks^16–18^, code converters^19–21^, sequential circuits^22–26^, memories^27–31^, etc., for different applications. Different types of systems and devices are designed using QCA. One of the devices which can be suited to this technology is the memory device. The device's data storage mechanism for data and information that allows retrieval (reading or writing) is called random access memory (RAM). The RAM is constructed in matrix like structure consisting of rows and columns and the process of writing and reading data in the RAM involves sequentially accessing and selecting certain elements within this matrix. To retain the data and information in the CMOS, a battery is mounted on the motherboard. The CMOS will wipe the data there whenever the battery is taken out or picked up on the motherboard^32–34^. Line-based and loop-based layouts are the two common ways that the QCA RAM can be implemented. In line-based RAM cell frameworks, the data moves in a straight path forward and backward^35^. Extra clock cycles are needed for line-based RAM circuit which complicate implementation.

The implementation of an efficient random-access memory (RAM) cell circuit can yield benefits such as reduced power consumption and improved performance. Consequently, the design of a cost-effective memory cell holds significant importance, as it serves as a fundamental building block for the entire RAM and is widely regarded as a critical component inside digital systems. Therefore, this paper presents the QCA design for the RAM cell, which is loop-based and single-layered.

### Paper contribution

The primary contributions of the work presented in this paper are:

- Fault Tolerant RAM cell based on MUX and Majority Voters in QCA is proposed.
- Fault analysis of RAM cell with single cell deletion, single cell addition and single cell displacement or misalignment defects is presented.
- Energy dissipation by the RAM cell is offered at different levels of kink energy.

## QCA fundamentals

### QCA cell

**Figure 1:**
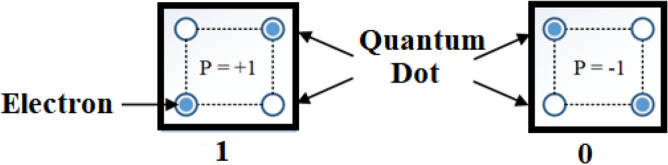
Depiction of QCA cell with its associated polarizations.

The QCA cell is the core unit of QCA technology. The polarization of a cell is determined by the position of its two electrons, which are depicted in Fig. 1 as − 1 (logical bit 0) and + 1 (logical bit 1), respectively^36^. The cell has two electrons and four quantum dots, and the two electrons can reside in any one of the four quantum dots. The electron is isolated and trapped in a specific area of space by the quantum dots, which function as energy wells. When quantum dots are in their regular, unexcited condition, the potential barrier prevents the electron from leaving the dots. When an electron is excited by the proper clock cycle, it accumulates energy and the potential barrier is lowered, allowing the electron to change states.

### Clocking in QCA

**Figure 2:**
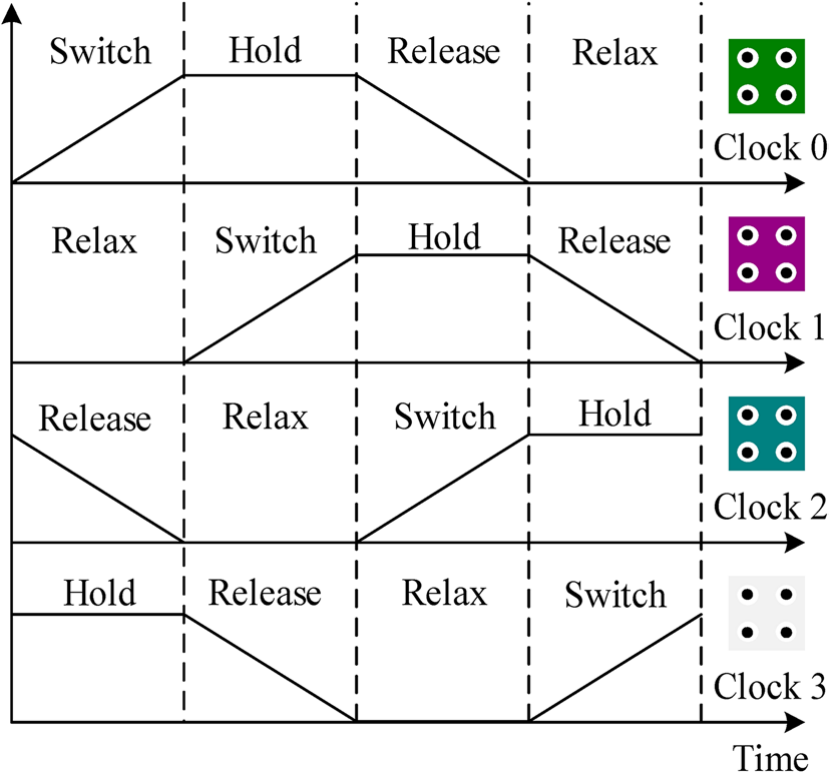
Clocking in QCA^37^.

Clocking in QCA is a critical operating factor. Cell polarization switching, data transmission via a QCA wire and logic computation in circuits is primarily clocking-based. Clocking determines a circuit's latency too. QCA clocking has four clocks, with each clock lagging by a 90º-phase the previous one, as shown in Fig. 2^37^. Every period of a clock has four parts: (1) Switch (2) Hold (3) Release (4) Relax. The height of the potential inter-dot barrier between the quantum dots describes the sections of the clock. When the height of the barrier is small, the electron gets stuck in the dot and cannot pass through the quantum tunnel. When the energy barrier lowers, the electron tunnels through the dot, and the cell switches the state. The phase difference allows data transmission through the wire by pipelining^36^.

### QCA logic gates

**Figure 3:**
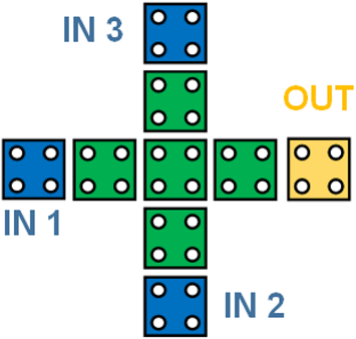
Input Majority Gate in QCA.

**Figure 4:**
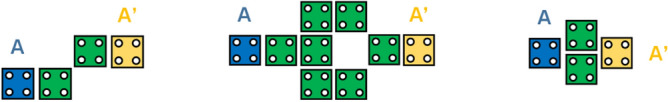
Various inverter configurations in QCA.

The logic gates which act as fundamental blocks in QCA are the majority gate and the inverter, as depicted in the Fig. 3 and Fig. 4. The basic equation of the three input majority gates is M(A, B, C) = AB + BC + CA. When one of the inputs is fixed as ‘1’, the majority gate operates as an OR gate else, it operates as an AND gate^7^.

### QCA Crossover

**Figure 5:**
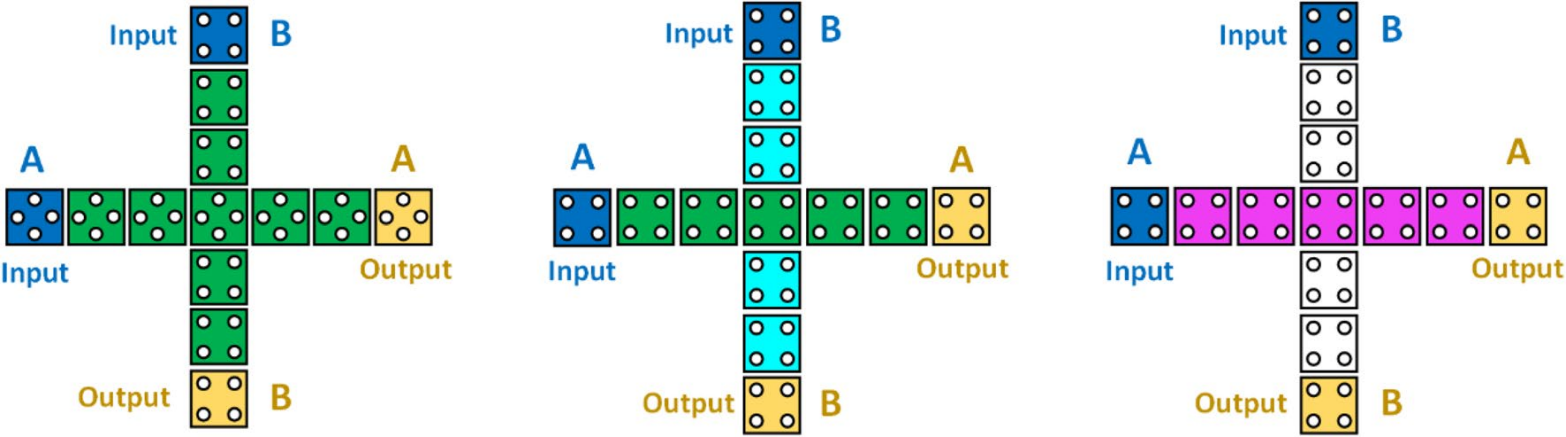
Depiction of coplanar crossovers in QCA.

**Figure 6:**
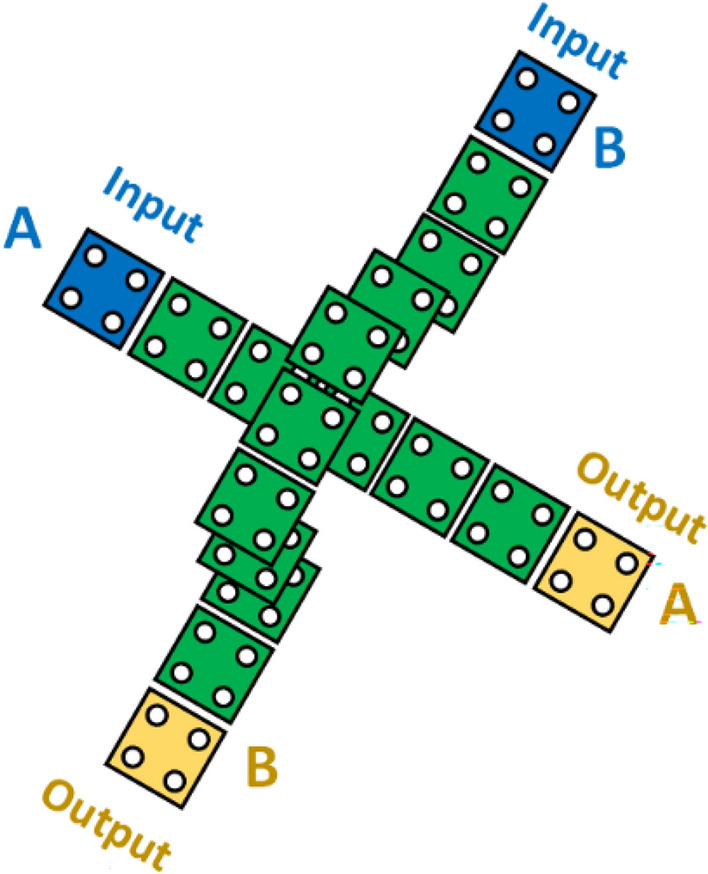
Multilayer crossover depiction in QCA.

Most often, we need to design complex circuits and designs wherein we need cross-overs to make the design less complex and in QCA, we have two main types of cross-overs viz coplanar crossover and multilayer based crossover. The former belongs to the single plane, as shown in Fig. 5, and the latter has more than one layer, as shown in Fig. 6^26,36^. The latter is complex yet needs less number of cells^8,38–40^. However, within the context of a fabrication situation, it is preferable for the components to be coplanar.

## RAM Cell Design

The unique characteristics of QCA, including its fast switching capability, regularity, and data retention ability in individual cells, render it a noteworthy tool for the construction of memory cells. Various designs of SRAM based on QCA have been proposed, with two primary approaches, namely loop-based and line-based, being often discussed across these designs. Clock zones are associated in the loop-based method to hold/retain data within a loop of the QCA cells. The cells in tandem forming a line are used in line-based RAM cell to store the previous value in it. Various techniques are utilized to design the memory cell in QCA technology.

**Figure 7:**
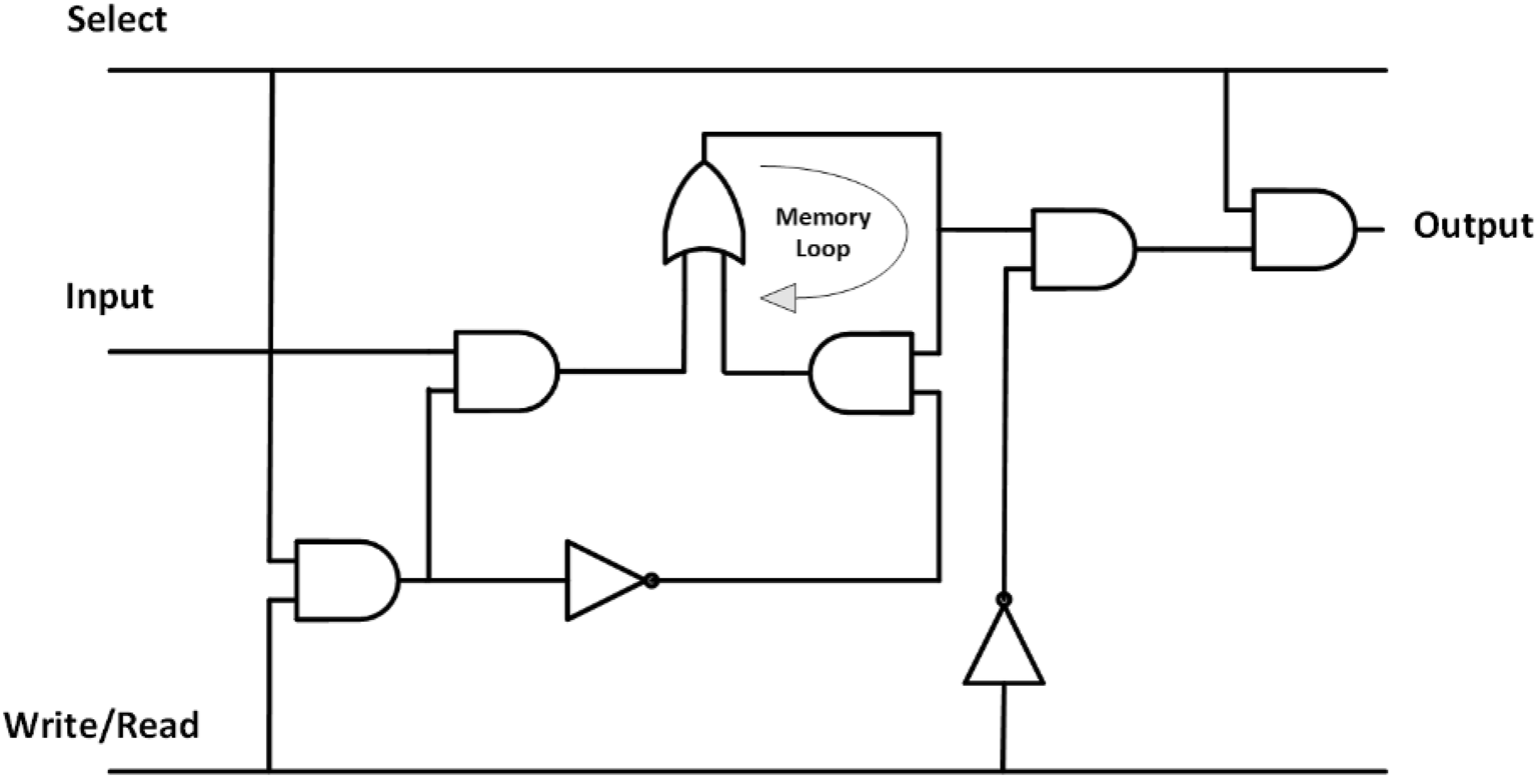
D-latch based conventional RAM cell.

The D-Latch is a fundamental component utilized in the creation of loop-based structures, which are frequently employed in the construction of RAM cells. In 2003, Walus et al. suggested using the D-latch as a RAM memory cell^41^, as displayed in Fig. 7.

**Figure 8:**
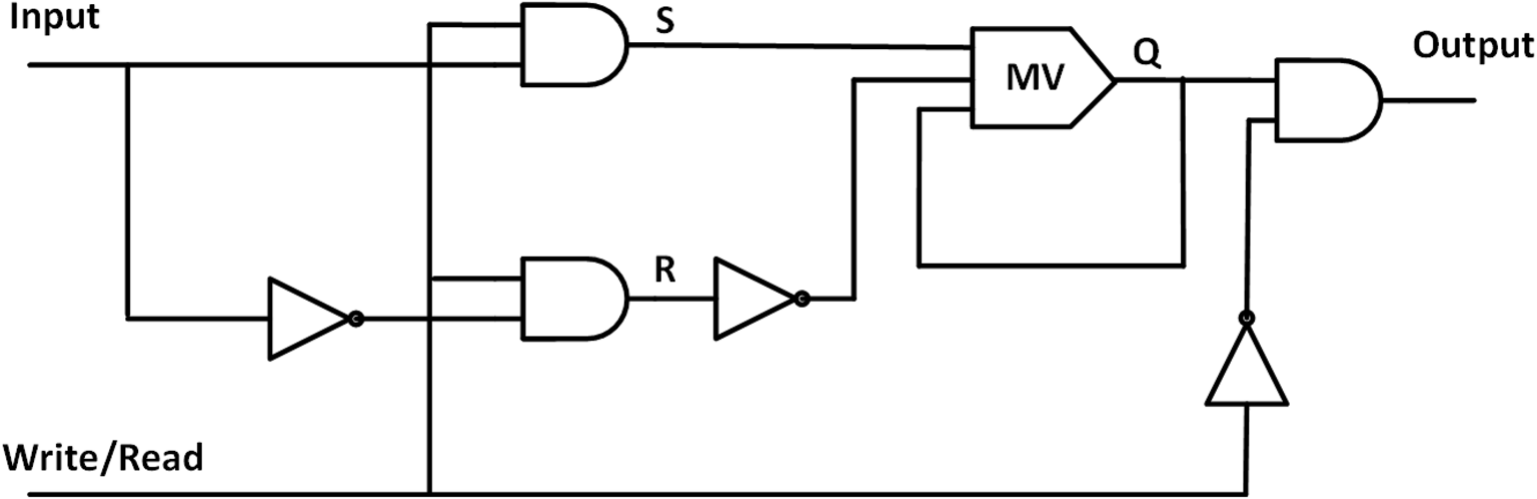
SR-latch based RAM cell.

Dekhordi et al. in^42^ proposed two SR-latch based RAM cells for the schematic shown in Fig. 8. The first layout having total cell count of 100, total area being occupied equal to 0.11 µm^2^ and having latency of 2, has the problem of unstable output same as that of the D-latch based RAM cell and the second design with regular clock zones was having the problem of large number of cells used, area occupied, lack of synchronization and unstable design. The proposed structure is comprised of two inputs and one output. When read/write = ‘1’, the input value is written in the output thus performing the write operation and when read/write = ‘0’, output path is opened and read operation is performed.

**Figure 9:**
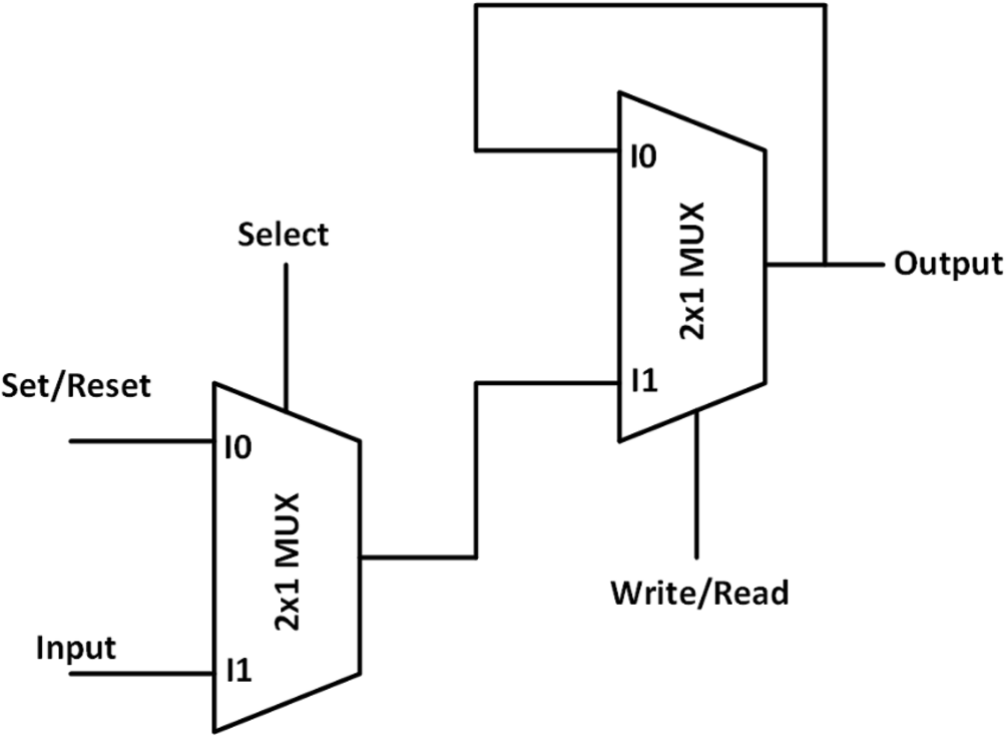
MUX based RAM cell.

Hashemi et al. ^43^ proposed the RAM cell using the 2 × 1 multiplexer. The schematic for the RAM cell based on this multiplexer is shown in Fig. 9. Irrespective of the values of *Select* and *Set/Reset,* when *Read/Write* = *‘0’*, the value of input cell is read and output does not change and when *Read/Write* = *‘1’,* and the *Select* and *Set/Reset* are ‘*0*’then the clear operation is performed and *Output* = *‘0’.*

The literature research reveals that there is still potential for further exploration and design of efficient and fault tolerant RAM cells. The current designs exhibit higher cell count, expanded area, and increased latency, resulting in high quantum costs.

### Proposed RAM cell in QCA

In our RAM cell, an efficient and fault tolerant 2 × 1 multiplexer ^44^ having three inputs, one fixed input and one output is used. The output equation of the multiplexer is given as:

1 $$Out\, = \,I_{0} Sel\, + \,I_{1} Sel$$

As per Eq. (1), when the *Select* line is ‘0’, then the value of *I*_*0*_ comes at the *output* and when *Select* line = ’1’, then the value of *I*_*1*_ comes at the *output*.

**Figure 10:**
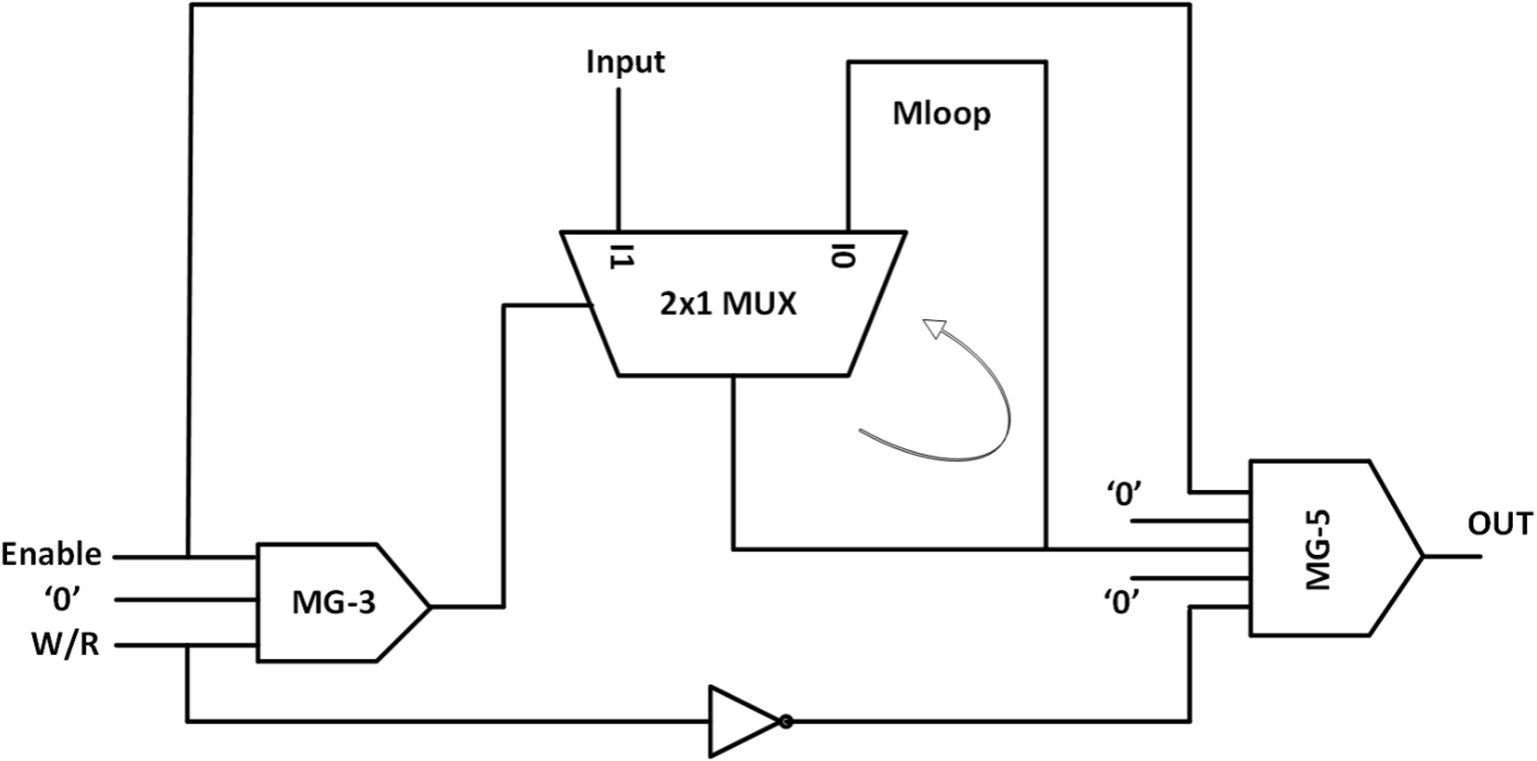
Schematic of MUX and Majority Voter based RAM cell.

Based on this multiplexer, we have proposed an efficient design of RAM cell which is having less cell count and area than the previous designs. The schematic diagram for the design is shown in Fig. 10.

**Figure 11:**
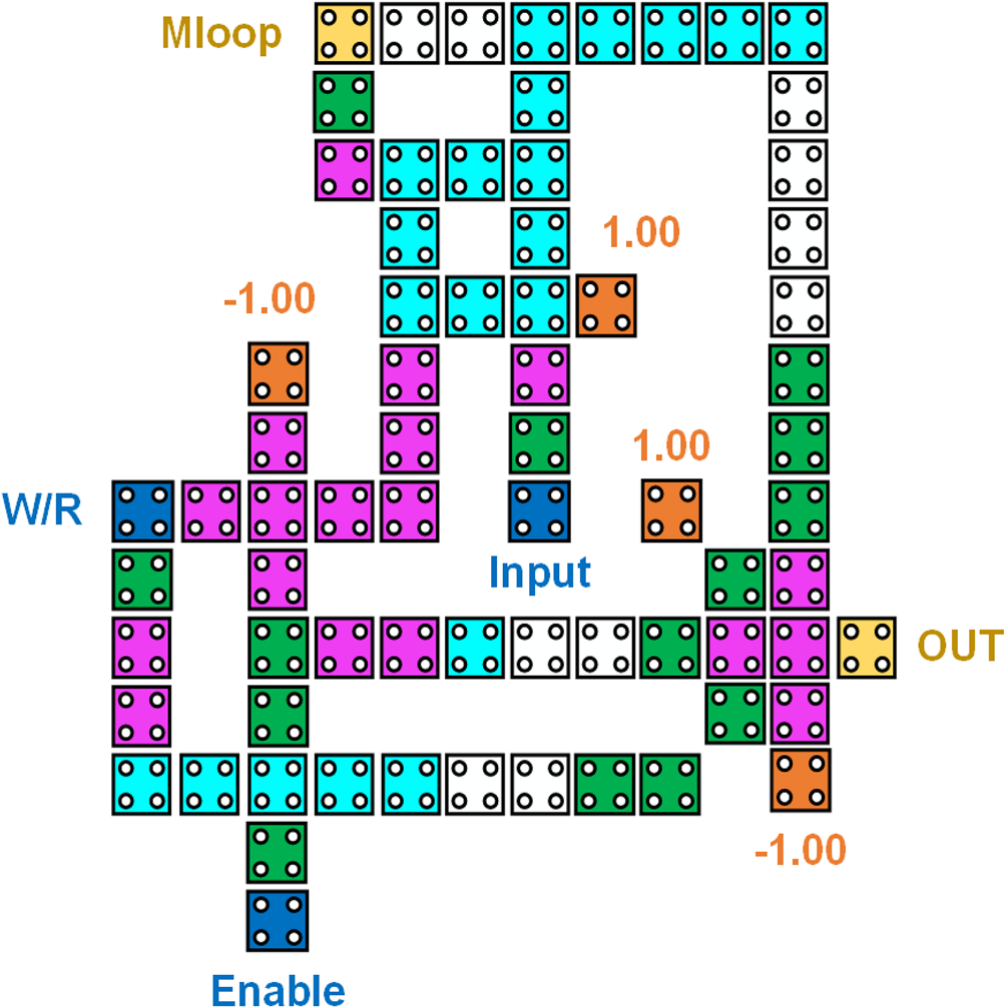
QCA design of proposed RAM Cell.

**Figure 12:**
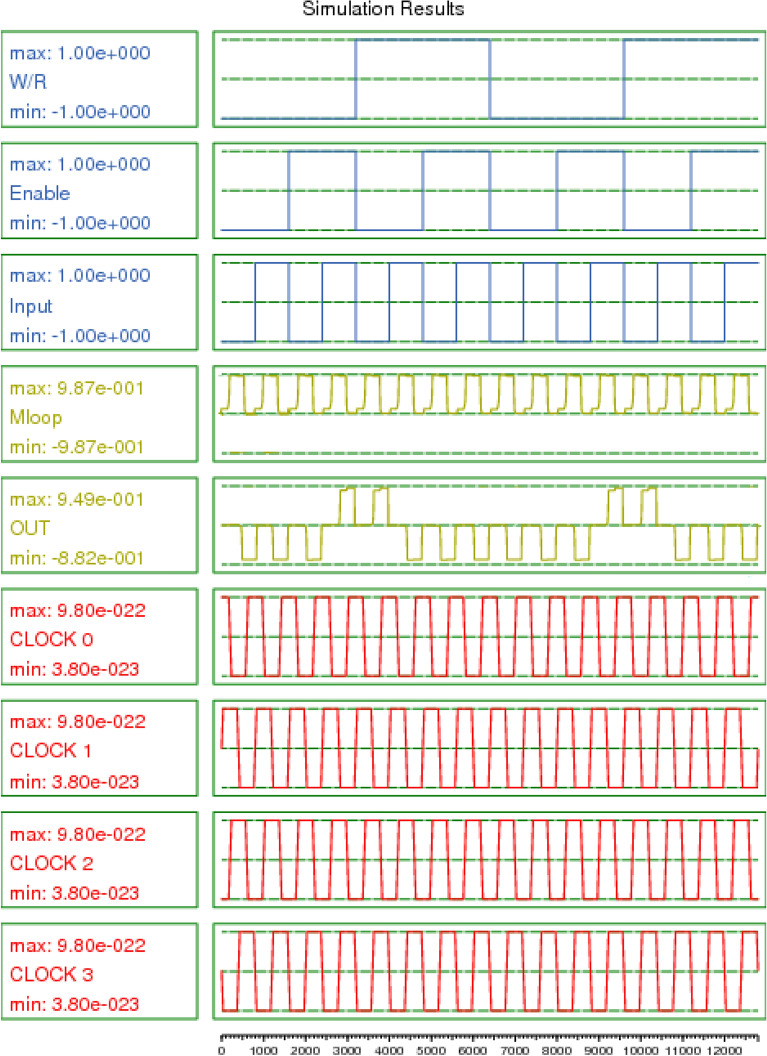
Output waveform of RAM Cell.

In addition to the 2 × 1 multiplexer, the proposed design also comprises of one 3-input majority gate and one 5-input majority gate. When the *Enable* input is set in ‘1’, write function is performed. Since two of the inputs of 3-input majority gate are ‘1’, thus the output of this majority gate will be ‘1’ and this output will be fed as input to the 2 × 1 multiplexer thus the value of the *input* will be written in Mloop and simultaneously transmitted to the 5-input majority gate along with the Enable signal. Now that the two inputs to this majority gate are fixed as ‘0’ and since *W/R* is ‘1’, the inverted signal would be ‘0’, thus giving the overall *output* = ‘0’ . The read function can happen by setting *W/R* = *‘0’* when the *Enable* = *‘1’* and thus using the first input of 2 × 1 multiplexer the stored data in Mloop can be easily retrieved through feedback and is thus obtained at the output. When the *Enable* input is set to ‘0’, the memory cell goes into the hold state. When the memory is in hold state, it keeps the information in the non-volatile memory unit i.e. Mloop. When *Enable* = ‘0’, the output of MG-3 becomes ‘0’ and thus value of Mloop will be transmitted to the first input of the 5-input majority gate (MG-5) and thus this leads to the holding of the content of memory. This is illustrated in Table 1. Here, in this design we have utilized the logical cross-over approach to get the desired operation. The QCA layout is in Fig. 11 and the simulation is shown in Fig. 12.

**Table 1:** Truth table of our RAM Cell.

Operation	Enable	Input	W\R	Mloop	Output
White	1	1	1	1	0
	1	0	1	0	0
Read	1	X	0	0	0
	1	X	0	1	1
Hold	0	X	X	Hold	0

## Fault tolerance analysis

The concept of fault tolerance in quantum-dot cellular automata (QCA) circuits pertains to the capacity of these circuits to maintain their proper functionality despite the occurrence of physical defects or errors resulting from imperfections in the fabrication process, environmental fluctuations, or other forms of interference. Some of the common defects/faults which occur in QCA structures are:

- Cell addition defect
- Cell omission/missing defect
- Defect due to misalignment of QCA cells

**Figure 13:**
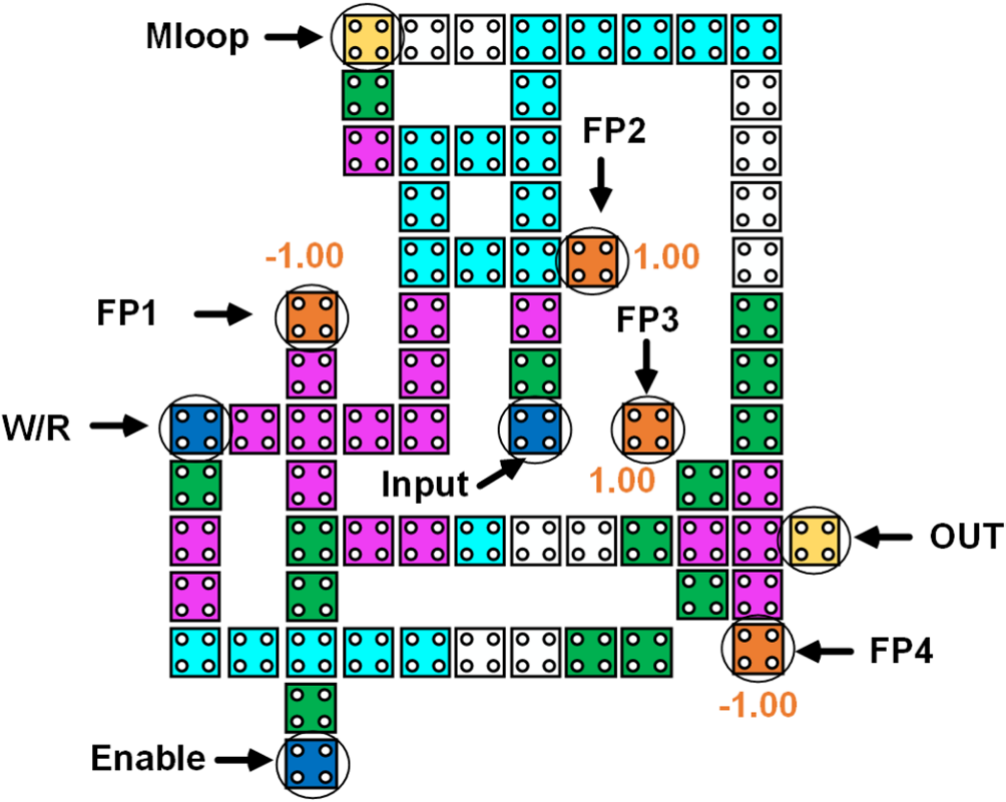
Defining the critical points for displacement fault testing in the proposed RAM cell.

**Figure 14:**
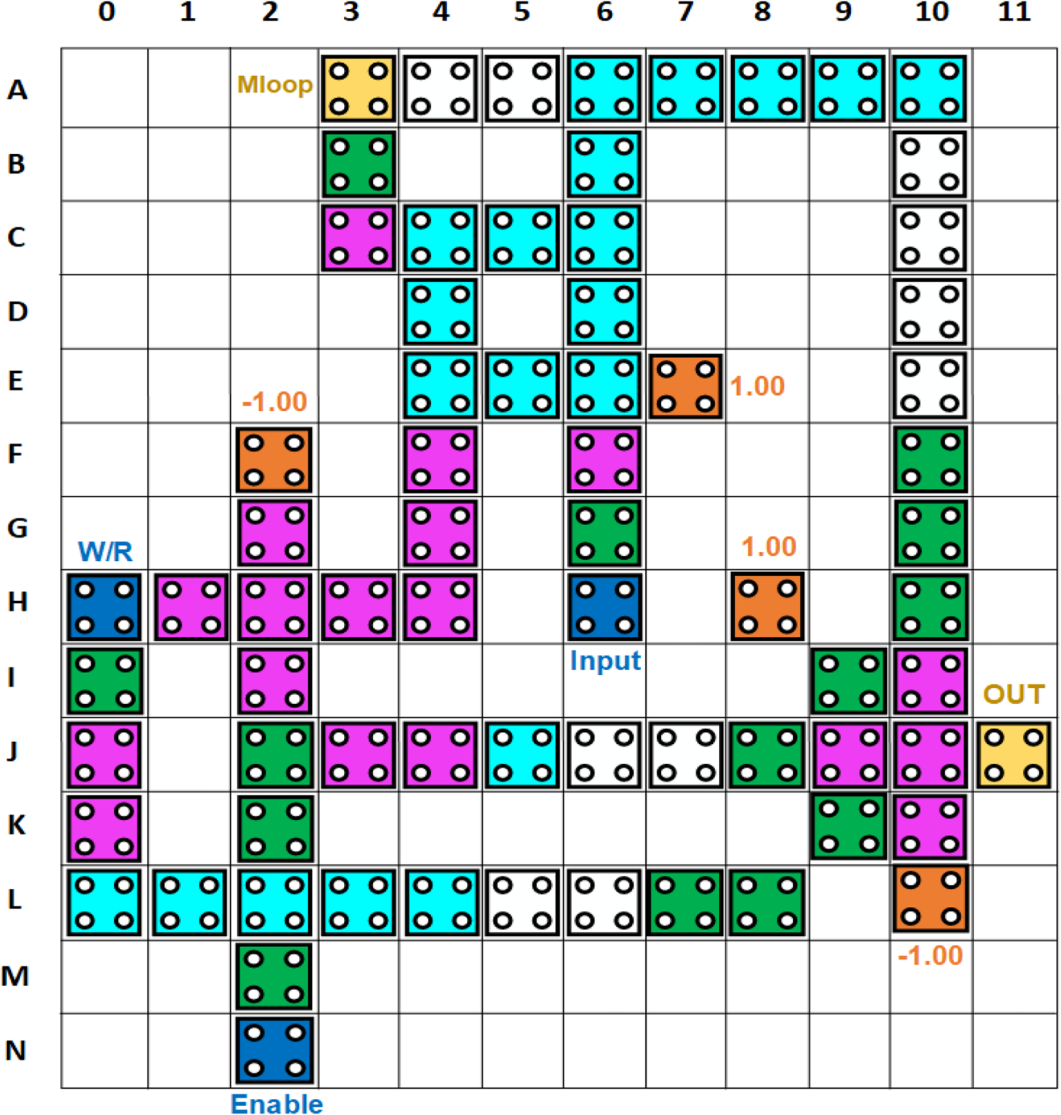
Grid Diagram of Proposed RAM Cell for fault tolerance analysis.

The above defects lead to the overall failure of the QCA system and in our proposed design we have selected some critical points which could possibly change or produce faults during the process of implementation of this design. Figure 13 shows the proposed design with specified critical points and Fig. 14 shows the grid representation of the RAM cell which is used to evaluate its fault tolerance. Each row and column are numbered for easy understanding. Table 2 shows the tolerance of the proposed design against the displacement of the specified cells (critical points) in each direction.

**Table 2:** Cell displacement (nm) error analysis of proposed RAM cell.

Analyzed cell	Displacement direction			
	West	East	South	North
Input	68	30	19	2
W/R	7	6	14	41
Enable	7	7	12	2
Mloop	3	6	4	4
OUT	4	5	4	4
FP1	∞	39	19	95
FP2	38	0	2	1
FP3	1	10	8	1
FP4	5	5	3	1

It is observed from Table 3 that the test vectors test vectors (0 0), (0 1), (1 0), and (1 1) have 3, 3, 1 and 1 faults respectively which leads to a total of 8 faults out of 92 tests performed. Also, the fault coverage by these test vectors (0 0), (0 1), (1 0), and (1 1) is 37.5%, 37.5%, 12.5% and 12.5% respectively. This leads to fault tolerance of (92—8) × 100/92 = 91.3% against single cell missing defect for the proposed RAM cell.

**Table 3:** Missing Cell Defect Analysis of proposed RAM cell.

Missing cell location	Test vector (0 0)	Test vector (0 1)	Test vector (1 0)	Test vector (1 1)
	*Expected Output * = *Simulated Output*	*Expected Output * = *Simulated Output*	*Expected Output * = *Simulated Output*	*Expected Output * = *Simulated Output*
B3, B6	Yes	Yes	Yes	Yes
C4, C5, C6	Yes	Yes	Yes	Yes
D4	Yes	Yes	Yes	Yes
D6	Yes	No	Yes	Yes
E4, E5, E6	Yes	Yes	Yes	Yes
G2	Yes	Yes	Yes	Yes
H1, H2, H3	Yes	Yes	Yes	Yes
H10	No	Yes	Yes	Yes
I2, I9	Yes	Yes	Yes	Yes
I10	No	Yes	Yes	Yes
J8	Yes	Yes	Yes	Yes
J9	Yes	No	Yes	Yes
J10	Yes	Yes	No	No
K9	Yes	No	Yes	Yes
K10	No	Yes	Yes	Yes

It is observed from Table 4 that the test vectors test vectors (0 0), (0 1), (1 0), and (1 1) have 4, 4, 3 and 3 faults respectively which leads to a total of 14 faults out of 72 tests performed. Also, the fault coverage by these test vectors (0 0), (0 1), (1 0), and (1 1) is 28.57%, 28.57%, 21.43% and 21.43% respectively. This leads to fault tolerance of (72–14) × 100/72 = 80.55% against single cell addition based defect for RAM cell.

**Table 4:** Additional cell defect analysis of proposed RAM cell.

Additional cell location	Test vector (0 0)	Test vector (0 1)	Test vector (1 0)	Test vector (1 1)
	*Expected output* = *actual output*	*Expected output * = *actual output*	*Expected output * = *actual output*	*Actual output * = *expected output*
B4, B5	Yes	Yes	Yes	Yes
C7	Yes	Yes	Yes	Yes
D3, D7	Yes	Yes	Yes	Yes
D5	No	No	Yes	Yes
E3	Yes	Yes	Yes	Yes
F5	No	No	Yes	Yes
G1, G3	Yes	Yes	Yes	Yes
H9	Yes	Yes	No	No
I1, I3	Yes	Yes	Yes	Yes
I8	Yes	Yes	No	No
I11	No	No	Yes	Yes
K8	Yes	Yes	No	No
K11	Yes	Yes	Yes	Yes
L9	No	No	Yes	Yes

## Discussion

The evaluation of the efficiency of the proposed designs involves a comparison of various factors, including the cell count, area utilization, latency, and quantum cost of the QCA circuit. The comparison between the suggested RAM and other alternatives is presented in Table 5, revealing that the proposed RAM exhibits characteristics of low area utilization, low latency, and low quantum cost. The quantum cost can be defined as the multiplication of the overall area and the square of the latency.

**Table 5:** Performance based comparison analysis of RAM cell.

RAM Design	#Cell	Cell based area (µm^2^)	Total area (µm^2^)	Latency	Quantum cost
^41^	158	0.0512	0.16	2	0.64
^43^	109	0.0353	0.13	1.75	0.398
^42^	100	0.0324	0.11	2	0.44
^45^	92	0.0298	0.10	1.5	0.225
^46^	88	0.0285	0.08	1.5	0.18
^29^	87	0.0282	0.12	1.5	0.27
^42^	63	0.0204	0.07	2	0.28
Proposed	71	0.023	0.06614	1.5	0.149

It is evident from Table 6 that performance improvement of quantum cost in the range of 17.22% to 76.72% has been attained by the proposed RAM cell.

**Table 6:** Quantum cost of our RAM Cell and existing.

RAM design	Quantum cost	Quantum cost of proposed design	Percentage improvement
^41^	0.64	0.149	76.72%
^43^	0.398		62.56%
^42^	0.44		66.14%
^45^	0.225		33.78%
^46^	0.18		17.22%
^29^	0.27		44.81%
^42^	0.28		46.79%

The analysis of energy dissipation by RAM cell has been performed using QCAPro tool ^47^ which uses approximation method to identify erratic energy cells in the design (if any). Using Hatree-Fork^48,49^ the Hamiltonian is shown in Eq. (2).

2 $$H=\left[\begin{array}{cc}\frac{{-E}_{k}}{2}{\sum }_{i}{C}_{i}{f}_{i,j}& -\gamma \\ -\gamma & \frac{{E}_{k}}{2}{\sum }_{i}{C}_{i}{f}_{i,j}\end{array}\right] =\left[\begin{array}{cc}\frac{{-E}_{k}}{2}\left({C}_{j-1}+{C}_{j+1}\right)& -\gamma \\ -\gamma & \frac{{E}_{k}}{2}\left({C}_{j-1}+{C}_{j+1}\right)\end{array}\right]$$

The power dissipated by a QCA cell per clock cycle is expressed as:

3 $${P}_{diss}=\frac{{E}_{diss}}{{T}_{cc}}<\left(\frac{\hslash }{2{T}_{cc}}{\overrightarrow{\Gamma }}^{ +}\right)\times \left(-{\overrightarrow{\Gamma }}_{N}^{+} tanhtanh \left(\frac{\hslash \left|{\overrightarrow{\Gamma }}^{ +}\right|}{{k}_{b}{T}_{cc}}\right) +{\overrightarrow{\Gamma }}_{N}^{ -}tanhtanh \left(\frac{\hslash \left|{\overrightarrow{\Gamma }}^{-}\right|}{{k}_{b}{T}_{cc}}\right)\right)$$

**Figure 15:**
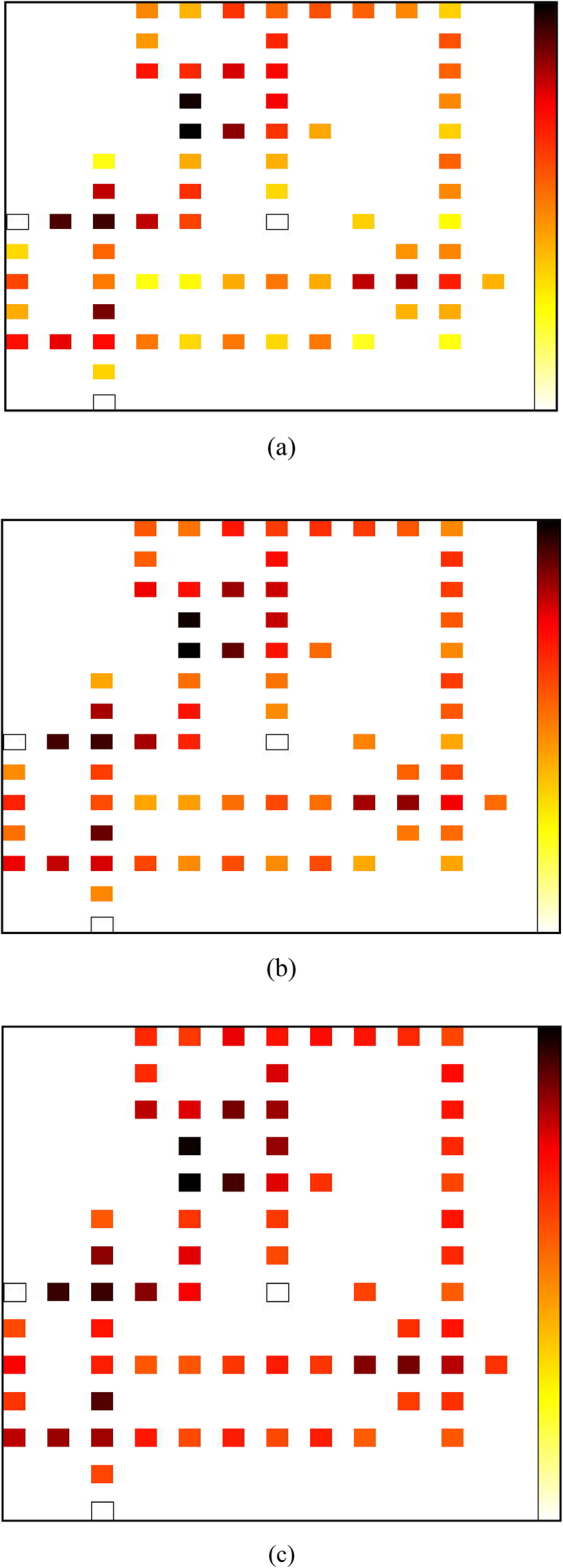
Energy dissipation map at (**a**) E_k_ = 0.5, (**b**) E_k_ = 1.0, and (**c**) E_k_ = 1.5 energy level of of proposed RAM cell at 2 K temperature.

The QCAPro tool provides the energy dissipation maps of the designs from which high energy dissipation cells can be identified and the design can be accordingly optimized to reduce the energy dissipation. Figure 15 shows the energy dissipation maps layout. It is evident that an increase in Ek levels results in a darkening of the cells, indicating that these dark cells exhibit the maximum energy dissipation among all cells in the design. The input and fixed polarization cells are depicted with white color in these maps.

Energy dissipation in QCA circuits arises from the electron transfer between quantum dots during state transitions, which facilitates the execution of logical processes. The kink energy levels are linked to the amount of energy needed for the reversal of polarization in adjacent cells inside a QCA cell. Kinks can be understood as borders that separate regions exhibiting contrasting polarization orientations within a given domain. The presence of higher kink energy levels in QCA circuits results in an increase energy barrier for phenomena such as kink switching and kink propagation. This phenomenon results in increased energy consumption during logic operations and clocking, hence reducing the energy efficiency of the circuit.

**Figure 16:**
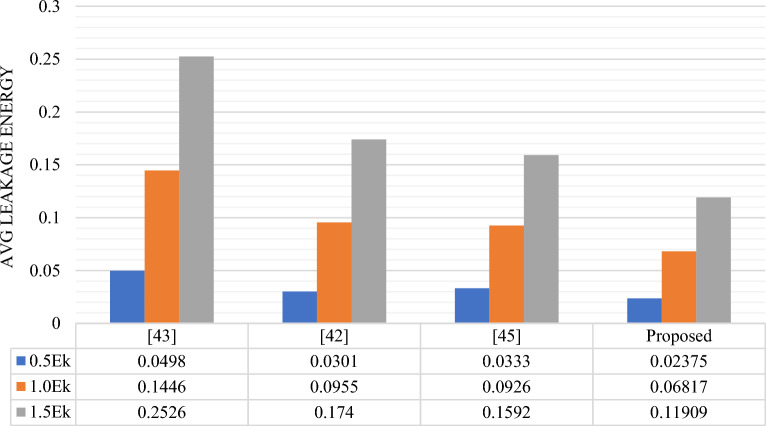
Avg. leakage energy (eV) dissipation comparison of different RAM Cells.

**Figure 17:**
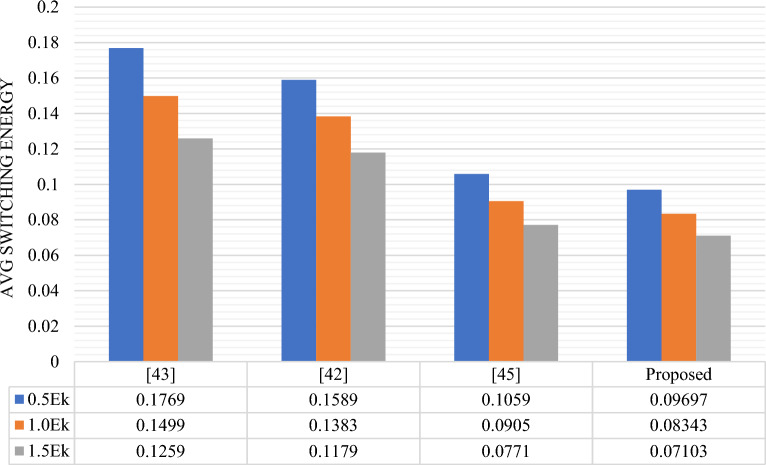
Avg. switching energy (eV) dissipation comparison of different RAM Cells.

**Figure 18:**
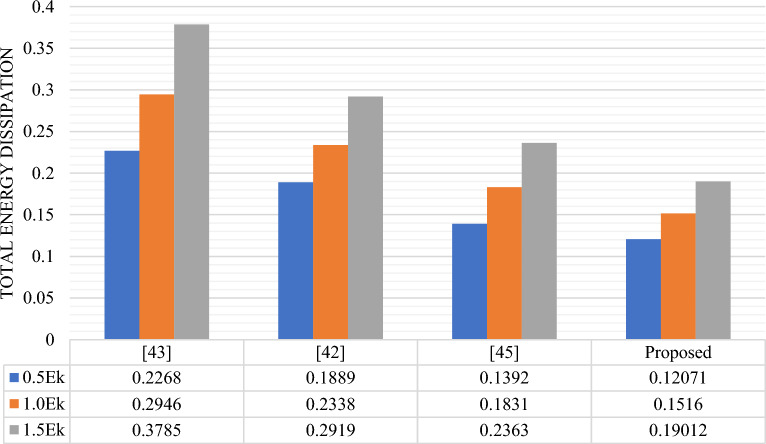
Total energy (eV) dissipation comparison of different RAM Cells.

The energy comparison of RAM cells is presented in Table 7 and graphically the average leakage, average switching and total energy dissipation comparison are shown in Figs. 16, 17 and 18 respectively. Based on the data presented in the table and graphs, it can be inferred that the proposed design exhibits the lowest energy dissipation across various kink energy levels. Consequently, this design appears to be more favorable for the development of efficient M × N RAM structures intended for low power applications.

**Table 7:** Energy dissipation analysis of RAM cells.

Structure	Average leakage energy dissipation (eV)			Average switching energy dissipation (eV)			Total energy dissipation (eV)		
	0.5 ${E}_{k}$	1 ${E}_{k}$	1.5 ${E}_{k}$	0.5 ${E}_{k}$	1 ${E}_{k}$	1.5 ${E}_{k}$	0.5 ${E}_{k}$	1 ${E}_{k}$	1.5 ${E}_{k}$
^43^	0.0498	0.1446	0.2526	0.1769	0.1499	0.1259	0.2268	0.2946	0.3785
^42^	0.0301	0.0955	0.174	0.1589	0.1383	0.1179	0.1889	0.2338	0.2919
^45^	0.0333	0.0926	0.1592	0.1059	0.0905	0.0771	0.1392	0.1831	0.2363
Proposed	0.02375	0.06817	0.11909	0.09697	0.08343	0.07103	0.12071	0.1516	0.19012

## Conclusion

This study introduces a novel design for a RAM cell utilizing a QCA architecture. The proposed design incorporates a 3-input and 5-input Majority Voter (MV) gate, in addition to a 2 × 1 Multiplexer (MUX). The QCADesigner tool was employed to validate the operation and behavior of the RAM cell, while the QCAPro tool was utilized to compute the energy dissipation of this RAM cell. Based on the evaluation of performance assessment, it can be inferred that the proposed design for the RAM cell exhibits efficiency when taking into account aspects such as cell count, area, and latency. Furthermore, it achieves a notable enhancement of up to 76.72% in terms of quantum cost. The fault analysis reveals that our RAM cell exhibits a fault tolerance of 91.3% and 80.55% when considering single missing cell and additional cell-based defects, respectively. Moreover, energy dispersal examination for various scenarios is likewise done and it is seen that the proposed configuration scatters least energy consequently making it more appropriate for designing low power applications. In future, this RAM cell can also be scaled to design M × N size RAM.

## Data Availability

The datasets generated during and/or analyzed during the current study are available from the corresponding author on reasonable request.
